# A Mosquito Inspired Strategy to Implant Microprobes into the Brain

**DOI:** 10.1038/s41598-017-18522-4

**Published:** 2018-01-09

**Authors:** Andrew J. Shoffstall, Suraj Srinivasan, Mitchell Willis, Allison M. Stiller, Melanie Ecker, Walter E. Voit, Joseph J. Pancrazio, Jeffrey R. Capadona

**Affiliations:** 1 Department of Biomedical Engineering, Case Western Reserve University, Cleveland, OH USA; 2 Advanced Platform Technology Center, Rehabilitation Research and Development, Louis Stokes Cleveland Department of Veterans Affairs Medical Center, Cleveland, OH USA; 3 Department of Bioengineering, The University of Texas at Dallas, Richardson, TX USA; 4 Department of Materials Science and Engineering, The University of Texas at Dallas, Richardson, TX USA; 5 Department of Mechanical Engineering, The University of Texas at Dallas, Richardson, TX USA; 6 Center for Engineering Innovation, The University of Texas at Dallas, Richardson, TX USA

## Abstract

Mosquitos are among the deadliest insects on the planet due to their ability to transmit diseases like malaria through their bite. In order to bite, a mosquito must insert a set of micro-sized needles through the skin to reach vascular structures. The mosquito uses a combination of mechanisms including an insertion guide to enable it to bite and feed off of larger animals. Here, we report on a biomimetic strategy inspired by the mosquito insertion guide to enable the implantation of intracortical microelectrodes into the brain. Next generation microelectrode designs leveraging ultra-small dimensions and/or flexible materials offer the promise of increased performance, but present difficulties in reliable implantation. With the biomimetic guide in place, the rate of successful microprobe insertion increased from 37.5% to 100% due to the rise in the critical buckling force of the microprobes by 3.8-fold. The prototype guides presented here provide a reproducible method to augment the insertion of small, flexible devices into the brain. In the future, similar approaches may be considered and applied to the insertion of other difficult to implant medical devices.

## Introduction

The parasitic bite of a female mosquito allows it to both inject an anticoagulant to thin the host’s blood, and then, like a miniature hypodermic needle, suck out blood to aid in egg production^1^. To enable the mosquito to penetrate the host’s skin with a set of blood-sucking needles (fascicles), multiple mechanisms are employed^2^. Specifically, the mosquito must increase the critical buckling force of each fascicle, while also reducing the force required to penetrate the skin.

A series of recent studies have discussed strategies taken by nature to prevent buckling and, in effect, improve the performance of percutaneous instruments (e.g., microneedles)^3^. Sakes *et al*. categorized strategies to either increase the critical buckling load, or conversely decrease the required penetration load. Interestingly, the mosquito does both, inspiring the design of “painless” microneedles^2,4^. We are particularly intrigued by the mosquito’s ability to increase the critical buckling force for adaptation to our application to implant intracortical microelectrodes into the brain. These devices offer a means of probing the functional neuronal network activity for both basic science and rehabilitation applications^5–7^.

**Figure 1:**
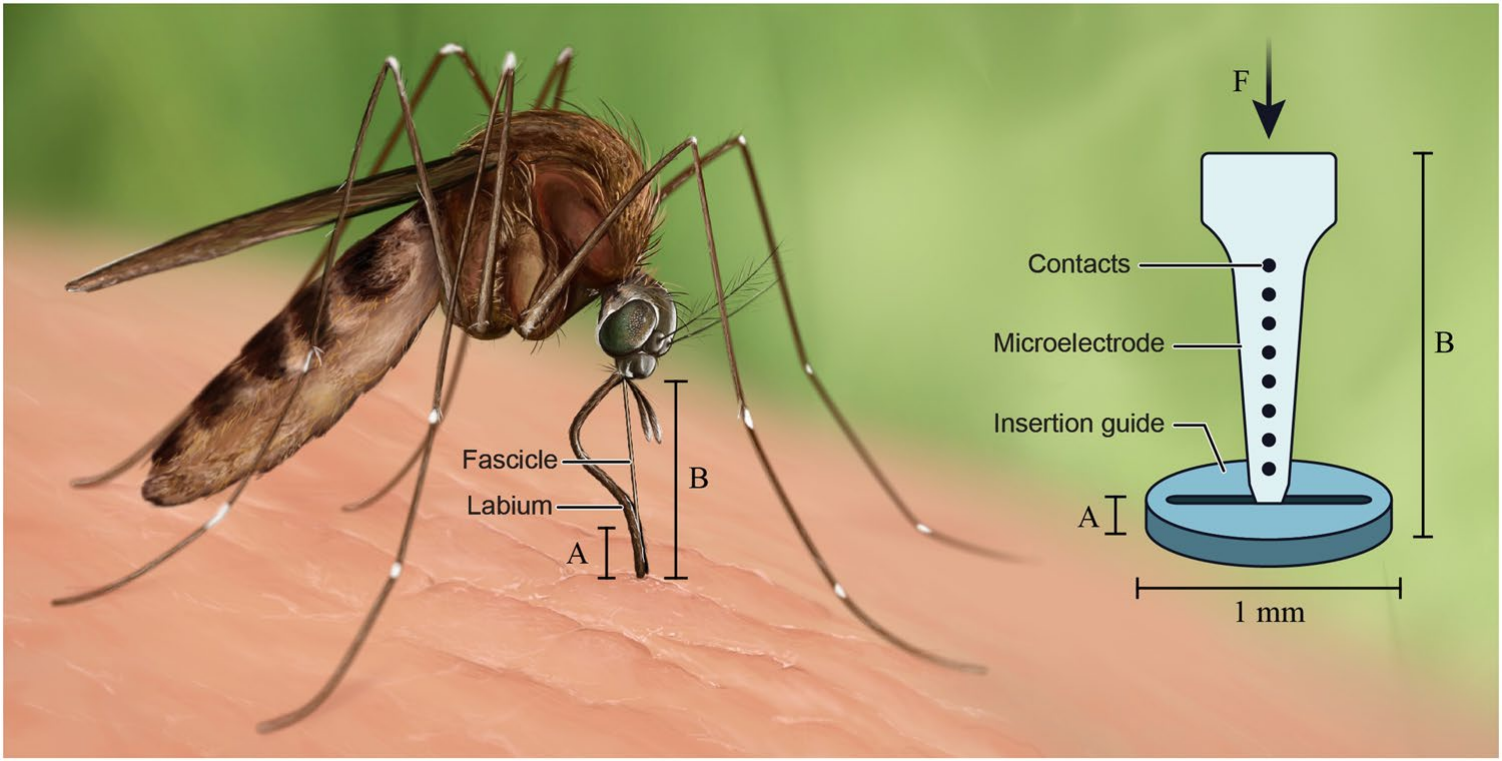
Mosquito-inspired guide to reduce buckling of flexible microelectrodes during insertion into brain tissue. Mosquitos use their labium (labeled above) to brace the fascicle during insertion through the tough skin. Mechanically, this changes the end-condition of the fascicle from a free- to a fixed-end condition and reduces the effective length. Similarly, our manufactured guide may be placed on the skull above the site of device implantation. A narrow slit, slightly wider than the microelectrode provides lateral support. The additional bracing prevents buckling. Figure was prepared by Erika Woodrum of the Cleveland FES Center, a contributor, with permission granted for use.

To increase the critical buckling load, the mosquito reduces the effective length of its fascicles by using a second structure, the labium, as an insertion guide (Fig. 1, left)^2,4^. To prevent fascicle buckling during insertion, the critical load must be higher than the penetration load. The mosquito fascicle and host tissue interface does not perfectly follow Euler’s formula for critical buckling load of an ideal beam due to complementary mechanisms of insertion (violating both the static condition and rigid-beam condition). However, one of the takeaways remains true—the effective length of the implant dictates the critical load that it can withstand without buckling^4^. Length and end-conditions (combined to make up the denominator “effective length” or “KL”) play an important role, defining an inverse-squared relationship to critical buckling force such that a 2-fold reduction of effective length results in a 4-fold increase of Euler critical load, F_Euler Critical_ (Equation 1):

1 $${F}_{Eulercritical}=\frac{{\pi }^{2}EI}{{(KL)}^{2}}$$

where *E* is the materials modulus of elasticity, I is the area moment of inertia, K is the effective length factor, and *L* is the unsupported length of the column. Through the use of the labium, the mosquito effectively reduces the length of the load bearing portion of the fascicle, enabling insertion of the higher aspect ratio needles, where the needles would not otherwise have been able to penetrate the skin. Intrigued by this capability, and with the goal of creating a new system for the implantation of ultra-small, flexible microelectrode devices into the brain, we set out to investigate whether a biomimetic strategy would enable an effective insertion guide approach (Fig. 1, right). Our work demonstrates for the first time that an insertion guide inspired by the fascicle/labium approach of the mosquito enables reliable insertion of microprobes within the brain.

Microelectrodes implanted in the cortex of the brain have the potential to be used in a number of exciting new neuroprosthetic applications specifically to enable brain-computer interface (BCI) and brain-machine interface (BMI) approaches. Neuroprostheses have the potential to improve the lives of individuals with paralysis and limb loss by reducing the burden of injury and enabling more full and interactive lives^5,8–10^. Unfortunately, penetrating intracortical microelectrodes such as those used for BMI applications demonstrate poor chronic neural recording performance and reliability^11,12^. The loss in performance is characterized by increased electrode contact and tissue impedance, decreased signal-to-noise ratio, and ultimately the inability to record from sufficient numbers of neurons to allow for robust decoding algorithms^13^. Performance typically degrades over several weeks-to-months, and is thought to result from both electromechanical and host tissue response mechanisms^13^.

While the small size of microelectrodes minimizes, in part, the iatrogenic trauma to the brain during insertion, penetration of the brain tissue is still sufficient enough to damage the blood-brain barrier and initiate an immediate inflammatory response^14–16^. The resulting foreign body response yields encapsulation of the recording device and both physically and electrically isolates the device from the adjacent neurons^13,17^. The hypothesis that inflammation is a key mediator of device longevity has been supported by several key studies^13,17–22^. Therefore, many different designs for microelectrodes exist, with an ever increasing array of approaches to minimize both the injury from implantation, and the resulting neurodegenerative inflammatory response. Recent approaches have used smaller and smaller electrode designs as well as flexible materials to both minimize the microelectrode footprint and the resulting strain on the cortical tissue^23–36^. A challenge that arises, however, is that during insertion, the device must be stiff enough to pierce the brain tissue without buckling^13,37–40^. A number of innovative approaches to address the issue of buckling during insertion have been developed and has been recently reviewed^41^. Some of the reported approaches include coating microelectrodes with sacrificial polymers or coatings that dissolve away during or after insertion^42–47^, fast insertion speeds^48^, a variety of introducer designs^49–51^, and materials that dynamically soften after insertion^38,40,52–54^. We recognized that the mosquito has solved the problem of reliable insertion of microscale needles into host tissue. Therefore, we sought to leverage the strategy used by the mosquito to enable robust insertion of novel microprobes into the brain. Mimicking the function should prove to be more broadly applicable to the various microelectrode designs currently under investigation.

To begin, guides were cut from plastic sheets made in varying dimensions ranging from 3 mm to 15 mm in diameter and 1/16″ to 1/8″ thickness using a laser cutter with motorized programmable x-y controls (150-Watt CO2 laser cutter, Universal ILS12.150D, Scottsdale, AZ). CAD drawings were created in CorelDRAW x6 (Ottawa, ON). During the optimization of the laser cutting process, we found that the following power settings to produce the highest quality cuts (achieving a balance between achieving the smallest kerf and least burnt edges possible): power (40%), speed (60%) and PPI (5000 dpi). A number of insertion guide materials were tested including low-density polyethylene (LDPE), high-density polyethylene (HDPE), polyethylene terephthalate glycol (PETG), polytetrafluoroethylene (PTFE) and poly (methyl methacrylate) (PMMA). We found PTFE and PMMA provided the best machinability with our laser, allowing thin and uniform slits to be cut. Further, PMMA had the best post-processing transparency, allowing the ready visualization of underlying blood vessels and other brain structures. Additional design features were also possible, including angled slits for inserting at non-90° angles, guides with breakaway perforations, and guides with circular holes cut into them instead of slits (as would be used for fiber optic cables or microwires). The guides were produced such that a microelectrode could be inserted through the slit with the use of handheld forceps or custom-fit stereotaxic frames (Supplementary Figure 2).

**Figure 2:**
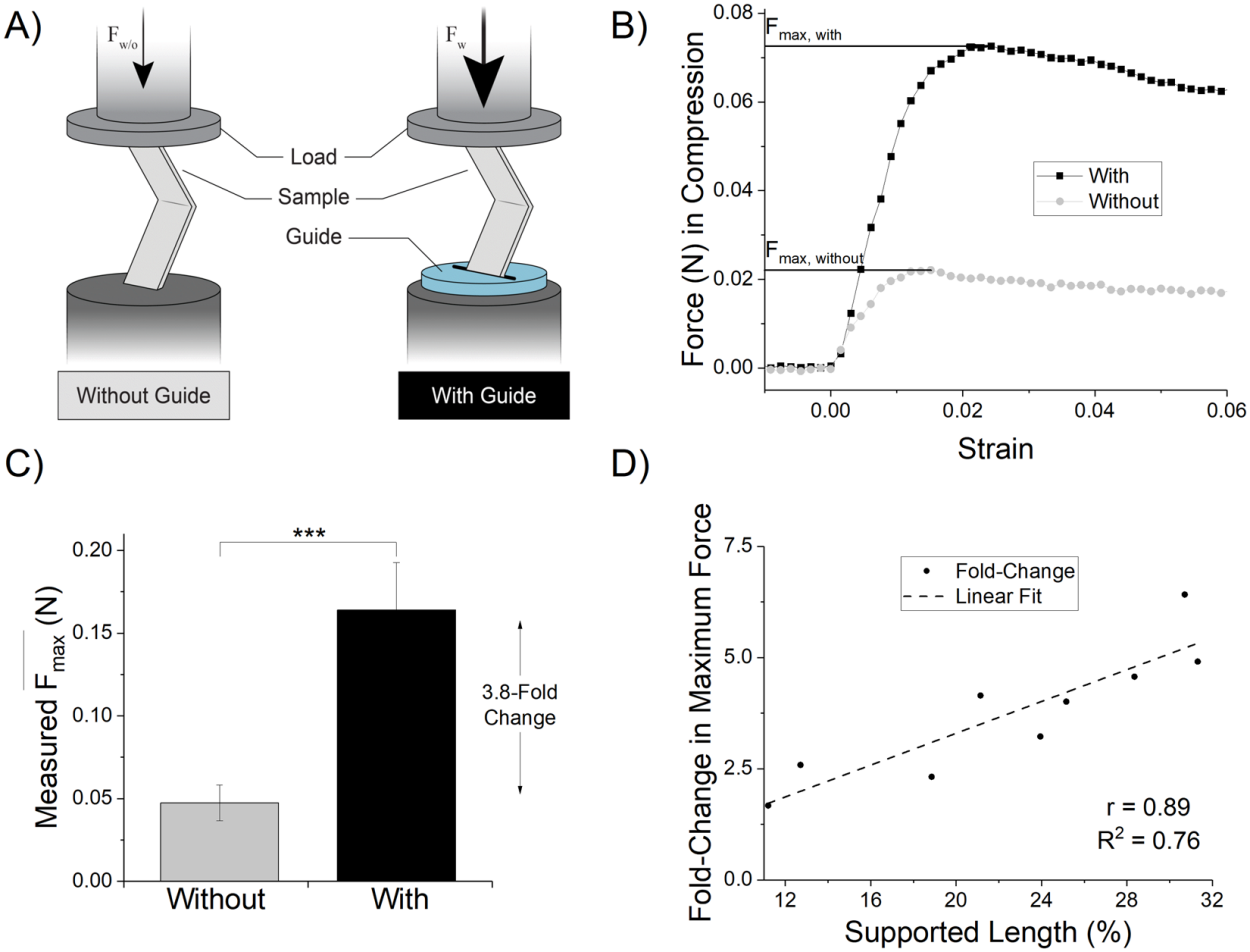
Mechanical testing of guide. (**A**) Illustration showing testing rig setup. Rectangular tests strips (n = 9) were placed in grips one side with a flat plate and force transducer opposing it. Maximal force in compression was measured with and without the guide in-place. (**B**) Representative mechanical testing traces for two back-to-back trials of the same dummy electrode. (**C**) Maximum force achieved in compression with and without the guide in-place, p < 0.001 (***), calculated by paired t-test of maximal achieved force with vs without guide in-place. (**D**) Scatterplot of fold-change in maximal force (i.e. fold-change = (F_max,with_/F_max,without_)) for each sample plotted against supported length (%) of the sample. Samples were of varying lengths, while the guide remained a constant 1/16′′ thickness. There is a very strong positive correlation as would be expected (i.e., the longer the probe, the more unsupported length there is to buckle). Part A of the figure was prepared by Erika Woodrum of the Cleveland FES Center, a contributor, with permission granted for use.

With prototype labium mimetic guides fabricated, we next tested the buckling mechanics using a linear actuator and a force transducer (Fig. 2A). Thin rectangular films (n = 9 samples) were compressed axially with or without the guide in place in a paired fashion (n = 18 individual trials) and maximal force was recorded. Since the 2 mm displacement was not sufficient to cause plastic deformation of the PE dummy samples, we judged that randomized-paired testing (with and without the guide) on each sample would be appropriate.

Peak force was achieved rapidly followed by a sudden decline and plateau in force, characteristic of buckling (Fig. 2B). We found that the guide increased the average maximally achieved force ($\overline{{F}_{max}}$, Equation 2) by 3.8-fold (±1.5 S.D.; Fig. 2C), and that the effect on force augmentation was correlated with the length of the microprobe (Fig. 2D).

2 $$\overline{{F}_{max}}=\frac{{\sum }_{i=1}^{n}({F}_{max})}{n}$$

The longer the microprobe, the less impact the 1/16′′ guide (~1.6 mm) was able to effect. If we limited the microprobe lengths to 5-times that of the guide thickness (or the guide being 20% of the microprobe length), the ratio of $\overline{{F}_{max}}$ increase then became 4.5-fold (±1.1). The $\overline{{F}_{max}}$ for all microprobes was 160 mN with the guide, compared to 50 mN without the guide (p < 0.001).

The critical design feature elucidated by this study was supported length, or the ratio of device length to guide thickness. This proportion was found to be largely determinant of maximal force before buckling. The design tradeoff of total device length versus desired depth of penetration is an important consideration. In this study, the depth of interest was set by the thickness of the rat cortex (~2 mm)^55^. Additional work is required to fully optimize and balance device length and cross-sectional geometry versus guide thickness. Furthermore, slit opening-thickness was minimized such that it was the smallest dimension possible, but still allowed the materials to slip between unimpeded. Operationally, this was approximately twice the film thickness (~150 µm). When testing, we found that a gap twice the thickness of the probe led to a good balance between easy slip between the two surfaces, without detrimentally impacting the bracing force of the guide. Tolerances 10–15 µm wider than the films themselves were too narrow and impeded insertion. Finer resolution laser cutting processes may enable a better optimization process to determine whether this gap thickness can be reduced further.

**Figure 3:**
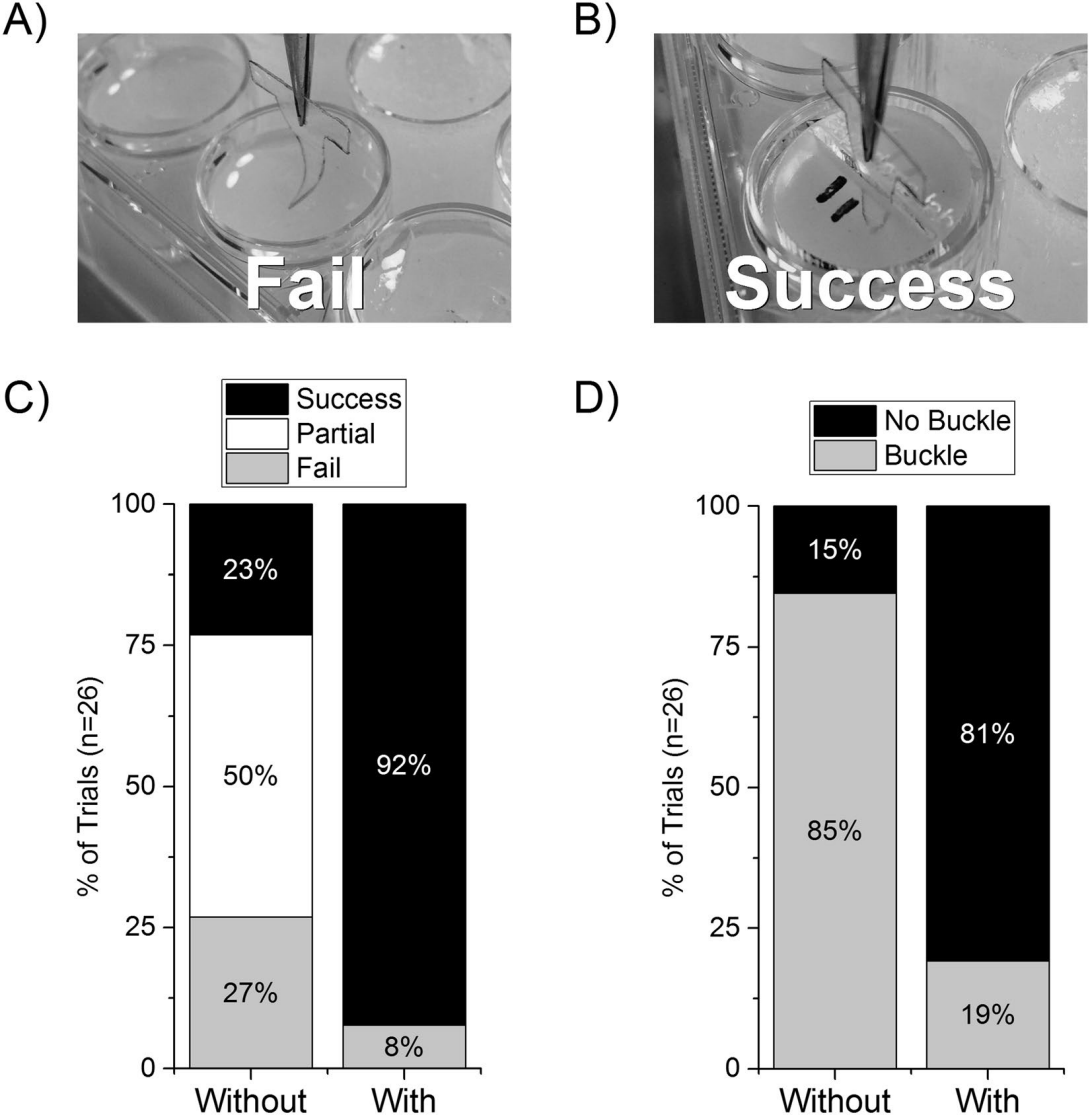
Agar gel model insertion with and without the guide. The microprobes were inserted using a stereotactic arm with a micropositioner. The microprobes were lowered to just above the surface of the gel, and inserted at a speed of ~1 mm/s. (**A**) Example of a failed insertion attempt without the guide in-place. Note the dummy microprobe buckling as it makes contact with the surface of the 0.6% agar model. (**B**) Example of a successful insertion with guide in place. (**C**) Successful rate of insertion with and without guides. (**D**) Rate of trials resulting in any buckling regardless of insertion status.

With an understanding of the design parameters between device and guide, we next developed a model of microprobe insertion with and without the guide, using 0.6% agar gel as a model for brain tissue^56^. Success was defined as complete insertion of the microprobe ~2 mm into the gel without any visible deformation of the microprobe; failure was everything else, including partial insertion or total deflection off the surface (Fig. 3A,B). Furthermore, insertion was still sometimes possible even in the event of buckling. However, buckling is potentially an undesirable outcome as it may mean the microprobe is then inserted at an angle and may not reach the desired brain structure target or perhaps be damaged. Interestingly, there was a marked difference in the number of successful attempts between those with and without the guide in place. The guide yielded successful insertion 92.3% of attempts versus 23.1% without the guide (Fig. 3C). Partial insertions, included as “failures” in the analysis, occurred 50% of the time without the guide and 0% with the guide (Fig. 3C). Moreover, with the guide, the dummy microprobes buckled only 19.2% of attempts versus 84.6% without the guide (Fig. 3D).

Encouraged by the substantial difference in implantation success rates achieved between conditions with and without the insertion guide, we next tested the impact of guided insertion on the implantation of intracortical microprobes into rat motor cortex. As mentioned above, many approaches have been developed to create intracortical microprobes that minimize implant mediated neuroinflammation^13^, including those that involve materials that dynamically soften upon insertion into the brain^39,40,57–60^. Dynamically softening materials, rely on a responsive stimulus such as moisture or body heat to effect the change in material properties after insertion^40^. Dynamically softening microelectrodes are typically polymer nanocomposites or shape memory polymers (SMPs), and are thus orders of magnitude softer than traditional materials used in microelectrode design. For example, polymer microelectrodes remain softer at room temperature (2–5 GPa) than typical silicon, tungsten, or Pt/Ir microprobes (150–500 GPa) (Table 1)^28,39^. Furthermore, the extremely small size of the devices makes the implantation procedure challenging such that an insertion guide strategy could minimize the likelihood of SMP microprobe buckling.

**Table 1:** Young’s moduli of example microelectrode substrates versus rat brain.

Material		Approximate Young’s Moduli (MPa)	Refs.
Silicon		~165,000	^61^
Pt/Ir (90%/10%)		~170,000	^62^
Cellulose nanocomposite	*(Pre/Stiff)(Post/Soft)*	4,2001.6	^40,63^
Thiol-ene/acrylateShape Memory Polymer	*(Pre/Stiff)(Post/Soft)*	~2,000~30	^28^
Rat brain tissue		0.015–0.45	^61,64,65^

While the methods and values vary in the reported literature, the relative magnitudes are conserved such that Young’s moduli of typical electrode substrates are much greater than polymer-based microelectrode substrates, even dynamically changing materials in their ‘stiff’ state.

In this study, fully softening thiol-ene SMP structures were fabricated similarly as previously reported^66^ so that the onset of their glass-transition temperature was just above that of body temperature and moisture-induced plasticization of the polymer network would cause softening after rigid insertion (Supplementary Figure 1). Therefore, as the microprobes were implanted and heated above their glass transition temperature (*T*_g_), the materials softened from ~2 GPa to ~30 MPa (indicated by vertical dashed gray line in Supplementary Figure 1).

**Figure 4:**
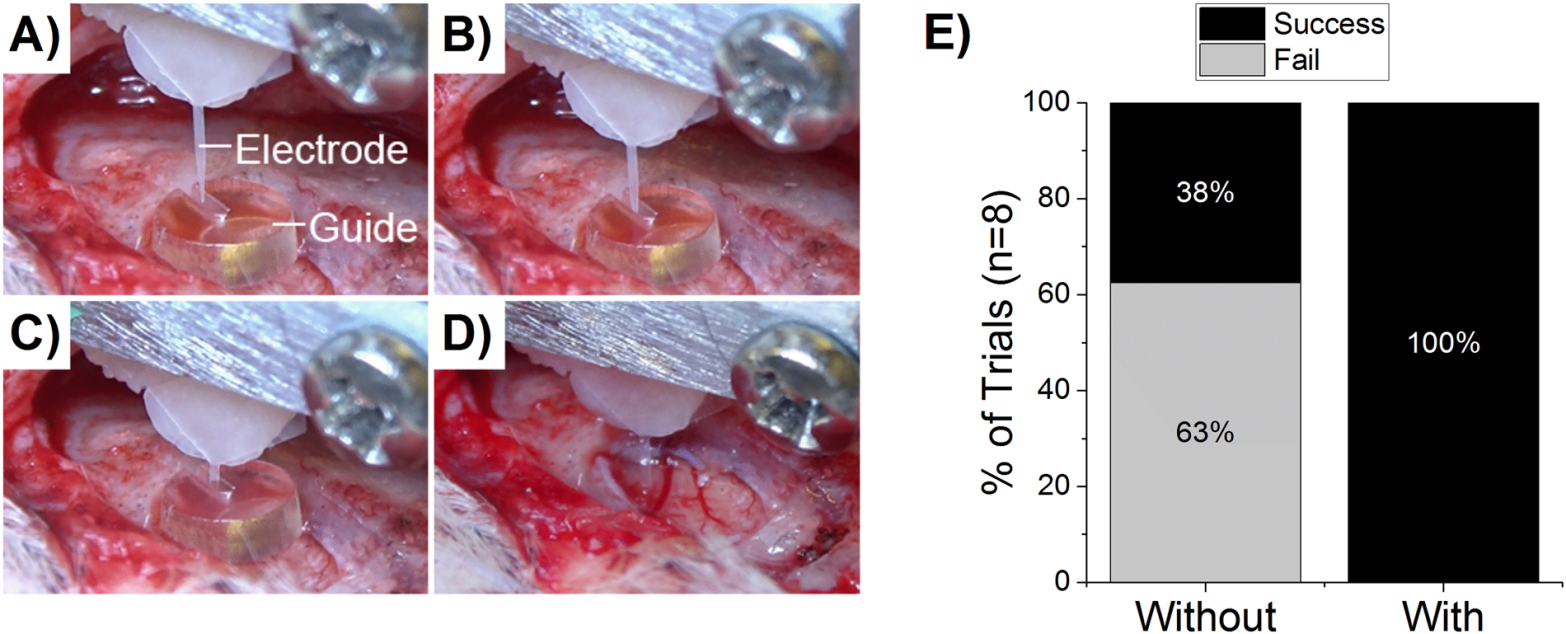
Implantation with shape memory polymer microelectrode with guide on brain surface. (**A**–**D**) feature progressive screen shots from a video taken during implantation of thiol-ene/acrylate microelectrodes. Guide thickness = 1 mm for reference. (**E**) 100% (8 of 8) insertion trials were successful with the automated insertion system and guide in place versus only 37.5% of trials without the guide.

While the system is designed to allow for successful insertion at room temperature, it was found that especially with an automated insertion system that controls the speed of insertion, the microprobes of chosen thickness were more prone to buckling, deflection and ultimately failed insertion without the use of an insertion guide (Fig. 4). With the insertion guide, we may be able to minimize device thickness in the future to achieve the same depth of penetration, leading to a less invasive implant.

We also examined the utility of the insertion guide in conjunction with the use of an automated motorized insertion system during *in vivo* implantations in the rat motor cortex (Kopf Instruments, Tujunga, CA, Model 2650 with a hydraulically driven micro-positioner). Due to the size of the guide, a large craniotomy was prepared (~1 cm^2^), allowing the entire guide to be placed directly in contact with the dura. The guides significantly increased the rate of successful insertion of SMP microprobes (p < 0.05). Specifically, the insertion guide enabled 100% (8 of 8 trials) successful insertion using the automated system compared to 37.5% (3 of 8 trials) without the guide (Fig. 4). It was noted in an early experiment that the guides were not as effective if placed on the skull, leaving an air-gap in which the microprobes were not supported at the site of entry into the brain (Supplementary Figures 3 and 4). Future design iterations may include either (1) a smaller diameter guide that doesn’t require such a large craniotomy, or (2) a beveled lip that allows the guide to be placed on the skull but with a recessed center which dips toward the brain surface.

Normally during intracranial microelectrode placement, the dura is reflected. As a demonstration of the added mechanical benefit to the insertion process, a dynamically softening SMP microprobe was inserted transdurally into rat brain (Supplementary Figure 5). Without the guide in place, buckling and failure to insert occurred every time. The microelectrodes (which are ~2 GPa at room temperature), buckled and deflected off the surface of the dura. With the guide in place, it became possible to insert the microelectrode through the tough dura. While it may still be preferred to reflect the dura to prevent shearing damage to the electronics on the face of the microelectrode, and to prevent an accelerated inflammatory response by dragging peripheral cells from the meninges into the parenchyma, insertion through the dura demonstrates the guide’s value as added lateral support to prevent buckling.

In summary, inspired by the labium guide of the female mosquito, we developed a novel method for introducing flexible microprobes into the brain. The bioinspired insertion strategy significantly reduces the propensity for buckling during insertion by increasing the insertion force without buckling by a factor of ~4. While this is a great improvement, it should be noted that the mosquito achieves much higher insertion efficiency by using a multimodal delivery system including: 1) barbed maxillae integrated in their labrum that oppositely reciprocate and saw open the skin and break the surface tension^2,4^, 2) an oscillatory insertion motion that results in a time-dependent shear thinning of the skin^3^, and 3) a follower-force applied by the labrum that further optimizes the mechanics to reduce buckling^4^. The reader is referred to the above-referenced sources for additional information regarding the mosquito bite mechanism.

While acknowledging that the mosquito has a much more sophisticated delivery system for microneedles, our analog guides are more easily fabricated from off-the-shelf polymer sheeting and are readily implemented during surgery. Furthermore, it was possible to use our bioinspired guide as a complementary strategy to dynamically softening polymers which are themselves a promising strategy utilized to overcome buckling of microelectrodes during insertion. Together, using the combined strategy, it was possible to: 1) insert flexible devices transdurally (e.g. without prior reflection of the dura) and 2) insert flexible microelectrodes into rat brain using an automated motorized insertion device. The purpose of these experiments was to establish the proof-of-concept data and identify critical design factors in terms of manufacturing feasibility, buckle-prevention efficacy, and intraoperative usability (Table 2). The approach can be further optimized by tuning the guide’s slit thickness and angle. While there are a number of other strategies to insert flexible electronics in the brain, the guide developed here is an additional tool and may be complementary to these other strategies. Finally, we envision the ease of implementation of our guide design to not just other microelectrode designs, but any other microscale medical device that suffers from difficulties in implantation.

**Table 2:** Critical Design Factors.

	Critical Design Factors	Recommendations
Manufacturing	Heat Resistant Polymers;Laser Cutting Parameters (Speed, Power);Small Geometry, Precise Cuts;Scalability	PTFE, PMMA;Fast speed, low power <100 µm features;100 devices in ~5 min
Mechanics	Supported Length;Slit Thickness;Guide Placement on Surface	>20% → >4x increase in F_max_;2x microelectrode thickness;Direct contact with dura
Usability	Visual characteristics;Placement and Removal Handling;Adherence to Surface During Insertion	Transparent materials;Design amenable to gripping tools;Thru holes for bonding to skull

A number of design factors were found to be critical to the manufacturability, mechanics, and usability of the guide.

## Methods

### Guide Design and Fabrication

Insertion guides were fabricated from various 1/16″ polymer sheets using a 150-Watt CO_2_ laser cutter (Universal ILS12.150D). The width of the channel at the top was ~150–200 µm, and narrowed to ~75–150 µm at the bottom. The laser cutting method was the fastest method at our disposal to develop a workable prototype. However, we envision several other potential advanced manufacturing methods to meet more repeatable and precise design criteria (e.g., lithography, chemical/photo-etching, and others). For the purpose of these experiments, 1/16″ polytetrafluorethyulene (PTFE) and poly (methyl methacrylate) (PMMA) were used. However, other thicknesses and materials could also be accommodated (e.g. polycarbonate, PETG, HDPE). Other incarnations of the device could include set non-90° angles to achieve specific targets or depths. The slit width and height may also be altered in order to accommodate electrodes of differing dimensions. Plastic sheets were ordered from McMaster Carr. Part numbers include: LDPE (8657K111), HDPE (8619K421), PTFE (8545K22), PETG (85815K11), PMMA (8589K11).

### Dummy Microelectrode Design, Characterization and Fabrication

For the purposes of testing the basic guide mechanics and insertion into agar gels, ‘dummy’ microelectrodes were fabricated from 3 mil (~75 µm) polyethylene (PE) films (Young’s modulus ≈300 MPa). PE closely resembles the flexible and compliant nature of conventionally used flexible microelectrodes. Different electrode dimension configurations were examined to produce an electrode for ‘dummy’ insertion testing. To exaggerate the buckling effects of the material during insertion, the length was made extremely long (in comparison to typical microelectrodes), with a length of 13.5 mm, base thickness of 6 mm, with a straight taper (internal angle 25°). They were fabricated using a 40 Watt CO_2_ laser cutter by placing the polyethylene films upon a sacrificial sheet of PTFE which absorbed the residual laser beam and heat. This ensured that the polyethylene was not burnt or singed during fabrication as the film tends to shrivel and form a blunt tip when heated to high temperatures.

Dynamically softening (dummy, non-functional) microelectrodes were provided by the Voit Lab (UT Dallas). The SMP-FS consists of a thiol-ene shape memory substrate composed of 0.5 mol% 1,3,5-Triallyl-1,3,5-triazine-2,4,6(1H,3H,5H)-trione (TATATO), 0.45 mol% trimethylolpropane tris(3-mercaptopropionate) (TMTMP), and 0.05 mol% tris [2-(3-mercaptopropionyloxy)ethyl] isocyanurate (TMICN). The SMP probes were prepared as previously described by radical initiated photo-polymerization^66,67^.

### Compression buckle testing

Compression buckle testing was performed using a universal testing apparatus (EnduraTec, Minnetonka, MN) equipped with a pneumatic linear actuator and load cell. Rectangular test strips (3-mil polyethylene film) were placed in clamps orthogonal to a custom-machined flat plate. The test strips, were advanced toward the flat plate either with or without the guide fixed in place to provide lateral support. Displacement was fixed to a maximum of 2 mm at a speed of 0.5–1.0 mm/s. Data was oversampled at 1000 Hz frequency to ensure capture of maximal forces attained during compression loading. Maximal force was determined with post-hoc analysis of the curves in Matlab (Natick, MA). Sample dimensions are shown in Supplementary Table 1.

### Gel Model Fabrication (Agarose Hydrogels) and Testing

Agarose hydrogels (0.6% w/v, UltraPure™ Agarose from ThermoFisher Scientific) were prepared as a model of brain tissue^56^. Agarose solutions were heated and poured into a Pyrex petri dish to cool and solidify at room temperature. The temperature will greatly impact the nature of the agar gel model and to a much lesser degree our test strip made of polyethylene which we selected for its mechanical properties at room temperature and which has a glass-transition temperature of Tg ~ −125 °C (significantly lower than both room and body temperature). All testing was performed at room temperature, for which the probe stiffness and agar gel model were optimized^56^. A stereotaxic micromanipulator was used to lower flexible dummy polyethylene electrodes into the agar gels with and without the guides in-place. The microelectrodes were lowered to just above the surface of the gel and the insertion guide was positioned accurately such that the microelectrode would enter and be guided into the agarose. Subsequently, the microelectrode was lowered into the hydrogel through the insertion guide at ~1 mm/s. The judgement of success was based upon a human-objective standard and included two facets: insertion and buckling. Insertion was successfully achieved if the microelectrode vertically entered the agar to a depth of 2–3 mm below the surface. Partial insertions occurred when the microelectrode penetrated the surface of the agar and subsequently buckled within the testing medium. In some cases, successful or partial insertions were achieved but after the electrode had already undergone some buckling, and were noted separately.

### Guide-Assisted Insertion of a Dynamically Softening Microelectrode

Rats were anesthetized with 5% isoflurane and given an intraperitoneal shot of a KXA (ketamine, xylazine, and atropine) cocktail as previously described^60^. The animal was then secured in a stereotaxic frame and then dexamethasone was administered subcutaneously. Lidocaine was then administered at the incision site. The scalp was shaved and the skin was removed from the skull area. Several craniotomies were created for the insertion tests. Insertion guides were fabricated from a clear plastic, PMMA with a diameter of 3 mm, a height of 1 mm, and a slit width of approximately 0.25 mm. The slit was accessible from the side of the device so that it could be removed after implantation. Implantation tests were completed using non-functional thiol-ene/acrylate SMP microprobes from the Voit lab (UT Dallas) with a 3 mm shank, approximately 200 µm width, and 30 µm thickness. Devices were manually positioned directly above the mosquito device slit. The micro-positioner motor drive was then activated to lower the device through the slit and into the brain at 2000 µm/s. All procedures and animal care practices were approved by, and performed in accordance with the Louis Stokes Cleveland Department of Veterans Affairs Medical Center Institutional Animal Care and Use Committees.

### Statistics

Peak force from compression testing was analyzed by paired t-test in Minitab (State College, PA). Statistics were run on comparison of the average maximal forces achieved by each sample with and without the guide in place (n = 9, displayed as fold-change in the graph, Fig. 2B). Percentage of successful trials (Fig. 3C), was analyzed with a 2 × 2 probability table (Outcome A: 1 = success, 2 = partial/failure; Group B: 1 = With Guide, 2 = Without Guide) with Fisher’s Exact test (n = 26 trials). Fishers Exact test and odds ratio 99% confidence interval were calculated in Matlab. Graphs were produced in Origin software (Northampton, MA).

## Electronic supplementary material

Supplementary Information
