# Supplementary material for: A Mosquito Inspired Strategy to Implant Microprobes into the Brain
Source: Sci Rep. 2018 Jan 9;8:122. doi: 10.1038/s41598-017-18522-4 (PMC5760625; doi:10.1038/s41598-017-18522-4)
Supplement: Supplementary file 1 — Supplementary Information [file 41598_2017_18522_MOESM1_ESM.pdf]

Supplementary information for:

A Mosquito Inspired Strategy to Implant Microprobes into the Brain

Andrew J. Shoffstall[1,2], Suraj Srinivasan[1,2], Mitchell Willis[1], Allison M. Stiller[3], Melanie Ecker[4,6], Walter E. Voit[3,4,5,6], Joseph J. Pancrazio[3,4,6], Jeffrey R. Capadona*[,1,2]

[1]Department of Biomedical Engineering, Case Western Reserve University, Cleveland, OH, USA;
[2]Advanced Platform Technology Center, Rehabilitation Research and Development, Louis Stokes Cleveland Department of Veterans Affairs Medical Center, Cleveland, OH, USA;
[3]Department of Bioengineering, The University of Texas at Dallas, Richardson, TX, USA;
[4]Department of Materials Science and Engineering, The University of Texas at Dallas, Richardson, TX, USA;
[5]Department of Mechanical Engineering, The University of Texas at Dallas, Richardson, TX, USA;
[6]Center for Engineering Innovation, The University of Texas at Dallas, Richardson, TX, USA;

*Direct correspondence to:
Professor Jeffrey R. Capadona
Case Western Reserve University
2071 Martin Luther King Jr. Drive
Cleveland, OH, 44107
Email: jeffrey.capadona@case.edu

Additional Background Information on Mosquito Bite Mechanics

Mosquitos use several mechanisms to reduce the force required to penetrate skin: maxillae that precede the needle and saw open the skin, and oscillatory (100-200Hz) motion during penetration.[1,2]. They further optimize the mechanics of penetration by using the labrum to support the fascicle to prevent buckling. Our analog guide primarily targets this third mechanism.

1) Maxillae: Mosquitos have two barbed maxillae integrated in their labrum that oppositely reciprocate and saw open the skin.[3,4] This, combined with lubricious secretions from the labrum, reduces the overall force required to penetrate the skin, down to an estimated 16.5 µN, which is several orders of magnitude lower than that measured from insertion of microneedles of similar size.[3,5] Depending on tip shape and microelectrode dimensions, required insertion forces have been reported between 5-50 mN.[6]
2) Oscillatory motion: The force of insertion force is further reduced via proboscis oscillation during penetration. The proposed mechanism is time-dependent shear thinning of the skin caused by vibration.[5] In their paper, Lee *et al*, found a 40% reduction in insertion force required, when oscillating at 500 Hz with a piezo motor in an axial orientation to the microneedle.[1] These results were consistent with previously published results from Yang *et al* demonstrating a greater than 70% reduction in penetration force with silicon hypodermic microneedle oscillating in the kHz range.[2]
3) Labrum support: The labrum supports the fascicle, such that the unsupported length is reduced. A study by Ramasubramanian *et. al.*, summarized the buckling mechanisms of the mosquito in great detail and explains that the mosquito's fascicle may indeed still buckle initially as it is inserted through the skin, and that both a tangential and follower force is applied to the end of the fascicle.[4] The following force increases the critical load by a theoretically calculated factor of 5, enabling the needle to penetrate the skin.[4]

Dummy Microelectrode Design, Characterization and Fabrication for Benchtop Studies

Polyethylene film (3 Mil) was used to mimic the flexible and compliant nature of conventionally used flexible microelectrodes. For Different sample dimensions were examined to produce an electrode for "dummy" insertion testing. For agar insertion testing, the length was made longer relative to typical microelectrodes so that the buckling was exaggerated and could be more easily visualized. Sample dimensions were 13.5 mm length, 6 mm base thickness, with a straight taper with an internal angle of 25°. Samples were fabricated using a 40W CO_2 laser cutter by placing the polyethylene films upon a sacrificial sheet of PTFE which absorbed the residual laser beam and heat.

For compression-buckle testing with the load cell setup, rectangular samples were prepared and characterized in table below for thickness (laser profilometry), length and width (digital calipers), and Young's modulus (DMA) **(Supplementary Table 1).**

Supplementary Table 1: Characterization of PE films used in compression buckle testing.

Measurement	Mean +/- S.D.
Profilometry<br>Thickness (µm)	70.3 +/- 1.5
Digital Calipers<br>Length (mm)<br>Aspect Ratio	7.9 +/- 3.3<br>2.1 +/- 0.8
Dynamic materials analysis (DMA)<br>Young's Modulus (MPa)	192.1 +/- 14.4

Additional Characterization of Thiol-ene Acrylate Shape Memory Polymer Dummy Microelectrodes (DMA)

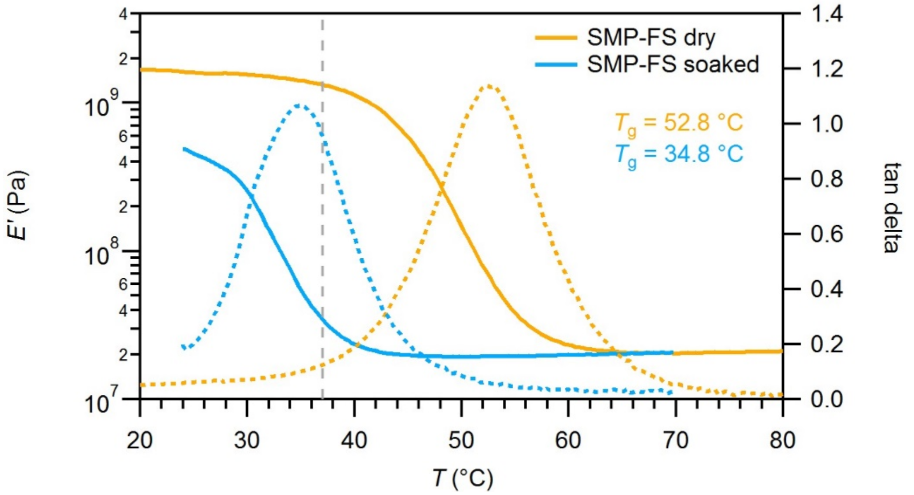

Supplementary Figure 1: Dynamic mechanic analysis of a fully softening SMP (SMP-FS) in the dry state (the condition before insertion; orange) and after being soaked for 1 h in PBS at 37 °C (mimicking *in vivo* conditions; blue). The peak of the tan delta (dashed lines), which gives the glass transition temperature (T_g) of the polymer, shifted from 52.8 °C to 34.8 °C due to plasticization.

Insertion of Thiol-ene Acrylate Shape Memory Polymer Dummy Microelectrodes into Rat Brains

- **Supplementary Figure 2:** Schematic representation of insertion using the guide 1) by hand, 2) using a stereotactic frame with manual micro-positioner, 3) using a stereotactic frame with a hydraulically driven micro-positioner.
- **Supplementary Figure 3:** Two different types of craniotomies used: small (2mm) vs large (5mm) such that the guide rests either on the skull or brain surface, respectively.
- **Supplementary Figure 4:** Insertion of dummy microelectrodes through circular PMMA guides with U-shape slit, resting on skull surface, using automated micromanipulator insertion technique.
- **Supplementary Figure 5:** Insertion through the intact dura using a rectangular PTFE guide resting on the skull surface, using by-hand insertion technique.

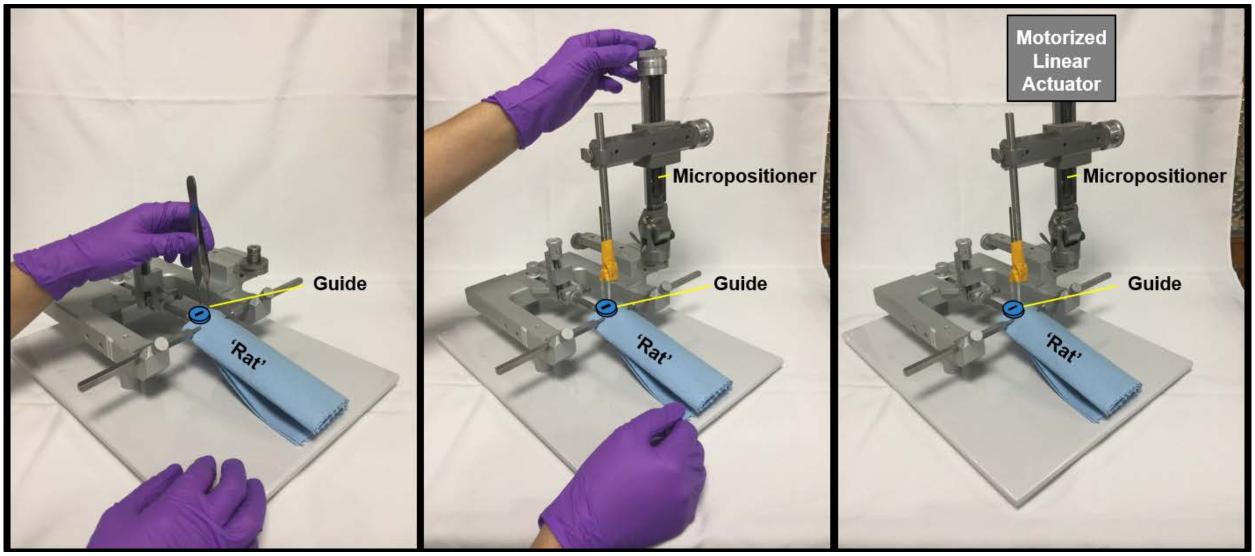

Supplementary Figure 2: Photographic representation of insertion using the guide 1) by hand, 2) using a stereotactic frame with manual micro-positioner, 3) using a stereotactic frame with a hydraulically driven micro-positioner.

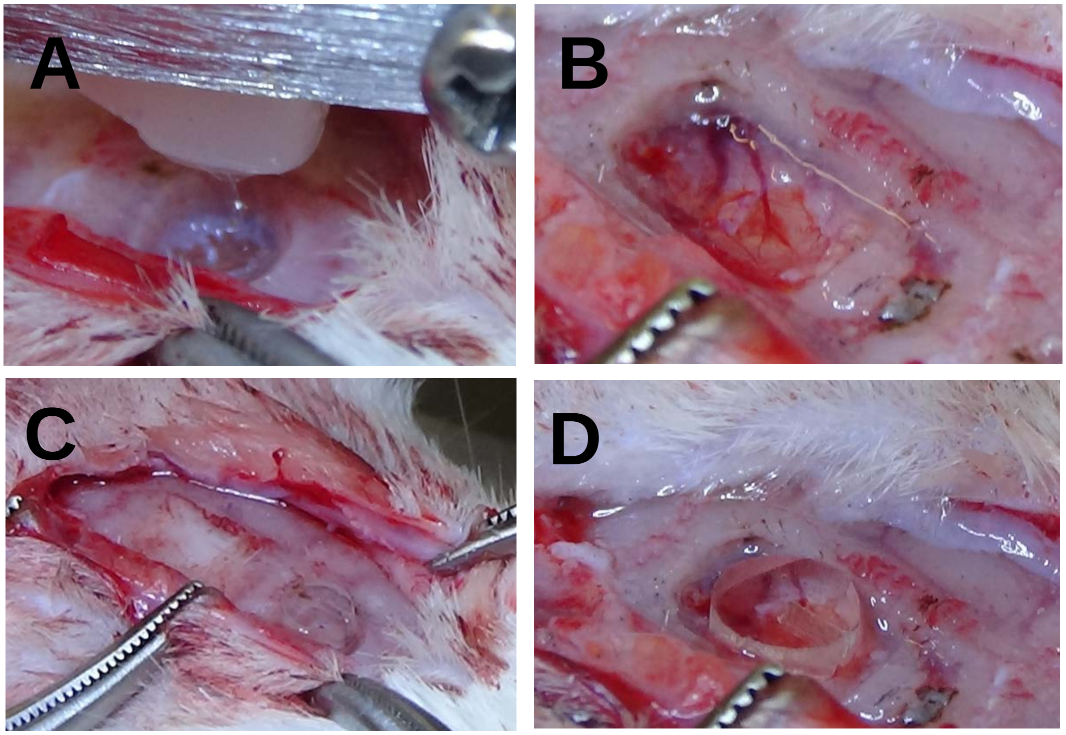

Supplementary Figure 3: Two craniotomy types were used for the insertion tests, with the dura removed from the brain surface in both cases. (A) During our standard intracortical surgeries, we use a small craniotomy (about 2 mm diameter). (B) To test with the guide sitting on top of the brain, a large craniotomy was used (about 5 mm by 4 mm), (C) The insertion guide sits on top of the skull, completely covering the craniotomy. (D) The insertion guide sits within the craniotomy, resting on the brain surface. Implantation device height = 1 mm for reference.

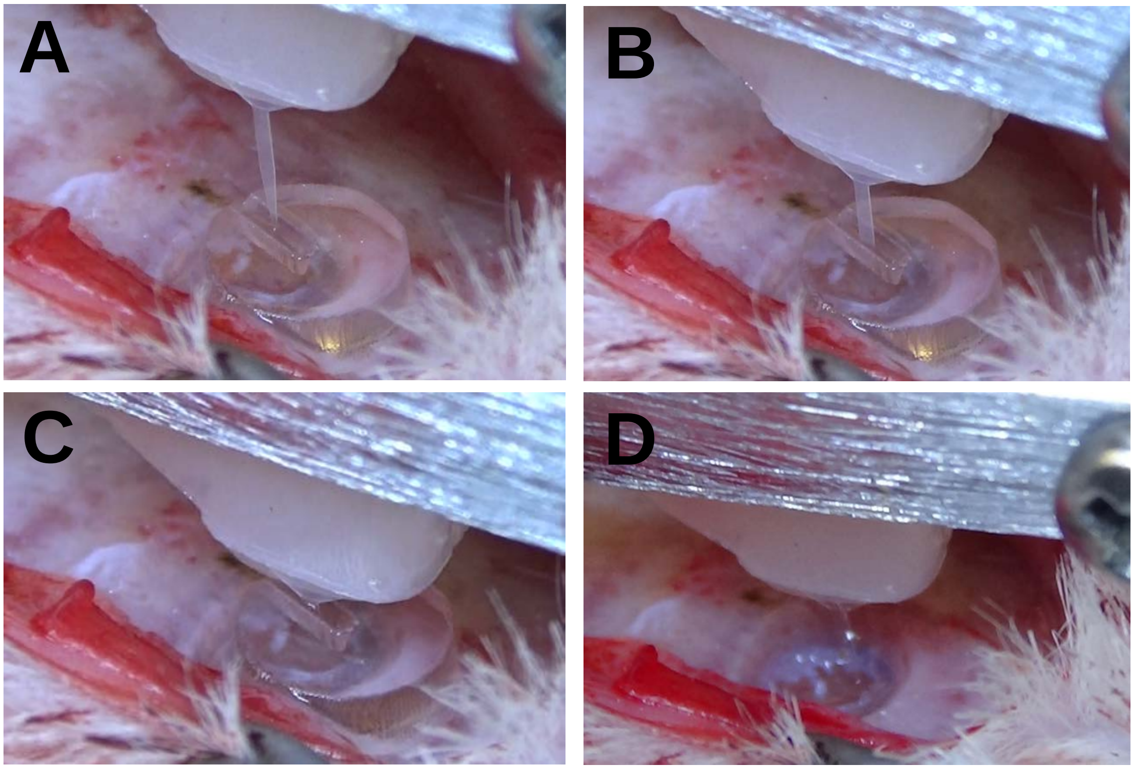

Supplementary Figure 4: Implantation with guide on skull surface using small craniotomy. Insertion guides were placed on top of the skull with the slit directly over the brain (with dura removed). A, B, C, and D feature progressive screen shots from a video taken during implantation. The biggest drawback with the small craniotomy is the low implant depth achieved due to the thickness of the skull and the guide. Using our automated system, all implants with the craniotomy were only implanted about 0.7-1mm depending on bone thickness in the craniotomy area. Implantation device height = 1 mm for reference.

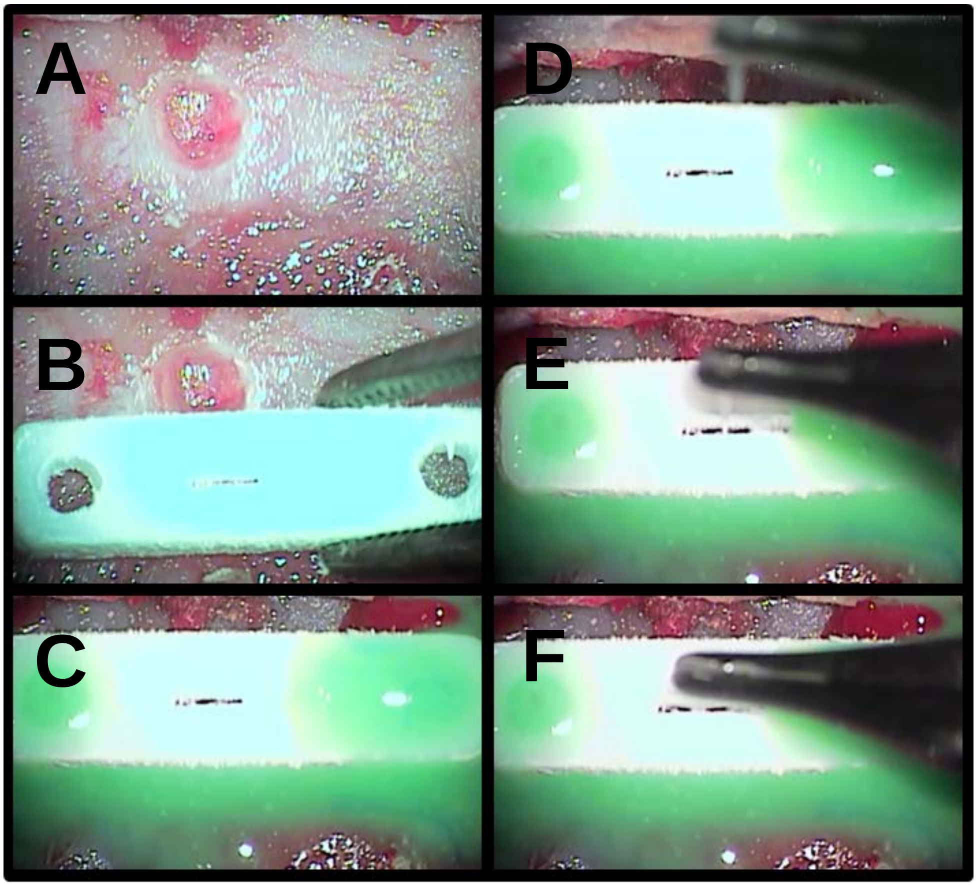

Supplementary Figure 5: The sequence of images shows the insertion of a flexible microelectrode dummy through the intact rat dura. The microelectrode dummy was made of thiol-ene acrylate shape memory polymer from the Voit lab (UT Dallas), thickness 30 micron, (A) Craniotomy performed, but leaving dura entirely intact, (B) Placing guide directly over the craniotomy, (C) Secured the guide in-place temporarily with green silicone elastomer, making sure to fill-in predrilled glue-holes on either side of the guide, (D-F). Translucent polymer electrode dummy piercing through intact dura.

References Cited:

1 Lee, F. W., Hung, W. H., Ma, C. W. & Yang, Y. J. Polymer-based disposable microneedle array with insertion assisted by vibrating motion. *Biomicrofluidics* **10**, 011905, doi:10.1063/1.49399481.4939948 [pii] 006698BMF [pii] (2016).

2 Yang, M. & Zahn, J. D. Microneedle insertion force reduction using vibratory actuation. *Biomedical microdevices* **6**, 177-182, doi:10.1023/B:BMMD.0000042046.07678.2e5277260 [pii] (2004).

3 Kong, X. Q. & Wu, C. W. Mosquito proboscis: an elegant biomicroelectromechanical system. *Phys Rev E Stat Nonlin Soft Matter Phys* **82**, 011910, doi:10.1103/PhysRevE.82.011910 (2010).

4 Ramasubramanian, M. K., Barham, O. M. & Swaminathan, V. Mechanics of a mosquito bite with applications to microneedle design. *Bioinspir Biomim* **3**, 046001, doi:10.1088/1748-3182/3/4/046001 (2008).

5 Sakes, A., Dodou, D. & Breedveld, P. Buckling prevention strategies in nature as inspiration for improving percutaneous instruments: a review. *Bioinspir Biomim* **11**, 021001, doi:10.1088/1748-3190/11/2/021001 (2016).

6 Fekete, Z., Nemeth, A., Marton, G., Ulbert, I. & Pongracz, A. Experimental study on the mechanical interaction between silicon neural microprobes and rat dura mater during insertion. *J Mater Sci Mater Med* **26**, 70, doi:10.1007/s10856-015-5401-y (2015).
